# Toward High‐Efficiency Solution‐Processed Planar Heterojunction Sb_2_S_3_ Solar Cells

**DOI:** 10.1002/advs.201500059

**Published:** 2015-04-02

**Authors:** Eugen Zimmermann, Thomas Pfadler, Julian Kalb, James A. Dorman, Daniel Sommer, Giso Hahn, Jonas Weickert, Lukas Schmidt‐Mende

**Affiliations:** ^1^ Department of PhysicsUniversity of Konstanz78457KonstanzGermany

**Keywords:** antimony sulfide, efficiency improvement, hole transport materials, simulations, solar cells

## Abstract

Low‐cost hybrid solar cells have made tremendous steps forward during the past decade owing to the implementation of extremely thin inorganic coatings as absorber layers, typically in combination with organic hole transporters. Using only extremely thin films of these absorbers reduces the requirement of single crystalline high‐quality materials and paves the way for low‐cost solution processing compatible with roll‐to‐roll fabrication processes. To date, the most efficient absorber material, except for the recently introduced organic–inorganic lead halide perovskites, has been Sb_2_S_3_, which can be implemented in hybrid photovoltaics using a simple chemical bath deposition. Current high‐efficiency Sb_2_S_3_ devices utilize absorber coatings on nanostructured TiO_2_ electrodes in combination with polymeric hole transporters. This geometry has so far been the state of the art, even though flat junction devices would be conceptually simpler with the additional potential of higher open circuit voltages due to reduced charge carrier recombination. Besides, the role of the hole transporter is not completely clarified yet. In particular, additional photocurrent contribution from the polymers has not been directly shown, which points toward detrimental parasitic light absorption in the polymers. This study presents a fine‐tuned chemical bath deposition method that allows fabricating solution‐processed low‐cost flat junction Sb_2_S_3_ solar cells with the highest open circuit voltage reported so far for chemical bath devices and efficiencies exceeding 4%. Characterization of back‐illuminated solar cells in combination with transfer matrix‐based simulations further allows to address the issue of absorption losses in the hole transport material and outline a pathway toward more efficient future devices.

## 1 Introduction

Research on new materials brought forth antimony sulfide (Sb_2_S_3_) as a promising candidate for efficient solution‐processible low‐cost solar cells. Sb_2_S_3_ is an abundant material,^1^ and has other favorable properties, such as a large intrinsic dipole moment,^2^ suitable optical band gap (≈1.7–1.9 eV),^3^, ^4^ a high dielectric constant for frequencies in the visible range (9.6–14.4),^5^ and a good band alignment in combination with various hole transport materials (HTMs).^6^, ^7^, ^8^, ^9^ While good band alignment is primarily beneficial for exciton dissociation and charge carrier transfer at the interface, a high dielectric constant as found in inorganic semiconductors such as Si or GaAs allows separation of charge carriers directly inside the bulk, provided that the crystallinity is high enough since exciton binding energies can be thermally overcome.^10^ In addition, a high absorption coefficient in the visible range (≈1.8 × 10^5^ cm^−1^ at 450 nm) allows the production of extremely thin absorber cells, which demand lower crystal quality, compatible with low‐temperature solution processing.^6^, ^7^ Thereby, external quantum efficiencies (EQE) of fabricated devices can exhibit values of more than 80% across the whole visible region.^2^

All these benefits led to the investigation of many different combinations of materials, morphologies, and deposition techniques for Sb_2_S_3_‐based cells to optimize performance and stability of fabricated devices. Among others, these investigations include ZnO and TiO_2_ as electron acceptors, both of which have been employed in flat junction devices and also as nanostructured electrodes.^2^, ^6^, ^7^, ^8^, ^9^, ^11^, ^12^, ^13^, ^14^, ^15^, ^16^, ^17^, ^18^, ^19^, ^20^, ^21^, ^22^ For the hole‐selective contact various HTMs have been employed, among them copper(I) thiocyanate (CuSCN),^8^, ^11^, ^13^, ^16^, ^20^ poly(3‐hexylthiophene) (P3HT),^6^, ^7^, ^12^, ^14^, ^19^, ^21^ poly[2,6‐(4,4‐bis‐(2‐ethylhexyl)‐4H‐cyclopenta[2,1‐b;3,4‐b′]‐dithiophene)‐alt‐4,7‐(2,1,3‐benzothiadiazole)] (PCPDTBT),^6^, ^18^, ^23^ 2,2′,7,7′‐tetrakis‐(N,N‐di‐p‐methoxyphenylamine)9,9′‐spirobifluorene (spiro‐OMeTAD),^2^, ^9^, ^22^ and liquid electrolytes.^17^, ^24^

**Figure 1:**
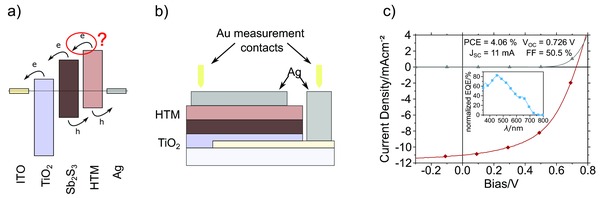
a) Illustrative energy level diagram of used materials, b) the schematic fabricated cell geometry, and c) the current density–voltage measurement of one of the most efficient devices. Corresponding external quantum efficiency of the device is shown as an inset in (c).

The use of additionally absorbing HTMs with matching energy band levels such as P3HT and PCPDTBT further promises increased light harvesting and higher photocurrents due to direct polymer contribution as in other hybrid solar cells.^25^ Such a photocurrent contribution comes into play via exciton generation in the HTM and subsequent exciton splitting via electron transfer from the HTM to the Sb_2_S_3_ (compare **Figure** **1**a). Therefore, interfaces become crucial and various surface modifiers, such as indium hydroxy sulfide (In‐OH‐S),^2^, ^13^ potassium thiocyanate (KSCN),^13^, ^14^, ^15^ lithium thiocyanate (LiSCN),^11^, ^16^ dipicolinic acid (DPA),^7^ and recently thioacetamide (TA)^18^ have been investigated to improve the interface between Sb_2_S_3_ and HTM. However, although P3HT and PCPDTBT exhibit matching energy band levels and already show a good bonding to Sb_2_S_3_ due to their thiophene rings,^6^ the electron transfer mechanism and the resulting contribution to charge generation by the HTM are still unclear. Even though this additional photocurrent contribution mechanism has been described in principle the extent to which it plays a role in real devices is yet to be clarified. In other hybrid systems where the polymer actively contributes to charge generation as, i.e., in hybrid solar cells utilizing a combination of the squaraine dye SQ2 and P3HT, this can be distinguished in the EQE, which unambiguously shows charge carrier contribution from both materials.^26^ Fine‐tuning of the interface properties further enhances the charge carrier and energy transfer between these materials, resulting in overall improved device performance.^25^

In contrast, EQEs of Sb_2_S_3_‐based devices do not show such a distinct contribution of both materials. Moreover, in combination with P3HT, fabricated solar cells even show a decreased EQE over the main absorption region of the polymer.^6^, ^7^ Even though this has been mentioned in a few previous reports, this issue has been widely ignored in the literature.^6^ The interaction of at least three mechanisms of the HTM can influence the EQE: (1) charge contribution of the HTM within its exciton diffusion length, (2) weak electronic bonding/unfavored band alignment at the interface of HTM and Sb_2_S_3_ leading to enhanced recombination, and (3) parasitic absorption of the bulk HTM that is not within exciton range to the interface. Thus, optimization of layer thicknesses, and surface modification are key aspects that have to be addressed to improve Sb_2_S_3_–polymer devices.

Both of these aspects have mainly been investigated on nanostructured cell geometries, which offer a drastically increased surface area and thus allow the use of extremely thin absorber layers. Such absorber‐sensitized high surface area structures are characterized by significantly enhanced device absorption, and require comparably lower Sb_2_S_3_ crystal quality due to resulting short path lengths for charge carriers. However, although a maximum power conversion efficiency (PCE) of about 7.5% could be achieved in Sb_2_S_3_‐based devices utilizing mesoporous TiO_2_ as an electron acceptor, thioacetamide as a surface modifier, and PCPDTBT as HTM^18^ in combination with an improved processing procedure, filling of nanostructures by Sb_2_S_3_ and subsequently by the HTM is challenging. This may result in voids and uncoated surface areas, which then can act as recombination centers. In addition, extremely thin Sb_2_S_3_ layers easily can form triple interfaces on nanostructured TiO_2_ due to a lattice mismatch between TiO_2_ and Sb_2_S_3_. This was demonstrated for chemical bath deposited (CBD)^2^, ^6^ and atomic layer deposited (ALD)^16^ Sb_2_S_3_ absorber films, where both techniques yield homogeneous coatings of amorphous Sb_2_S_3_ directly after deposition, but form bigger clusters and particles after annealing while the Sb_2_S_3_ de‐wets the TiO_2_. In combination with CuSCN as HTM this has been shown to result in enhanced charge carrier recombination at the TiO_2_–Sb_2_S_3_–CuSCN triple interface due to surface trap states in Sb_2_S_3_,^8^ and was confirmed by deep‐level transient spectroscopy also for PCPDTBT.^18^

On the contrary, a planar heterojunction geometry results in a less surface area demanding higher crystal quality of the consequently thicker absorber films but offers several other advantages. In particular, a planar heterojunction geometry, in general, needs less processing steps, is easier to upscale, and allows a higher reproducibility due to its simplicity. Furthermore, it allows an easier distinction between different layer contributions, which is a prerequisite for the separate optimization of individual solar cell components. In the new class of lead halide perovskite based solar cells, which analogous to Sb_2_S_3_ exhibit high dielectric constants for frequencies in the visible range and a good crystallinity^27^, ^28^ compared with common organic absorbers, fabricated planar heterojunction solar cells have been shown to perform, as well as their structured counterparts.^27^, ^29^ Furthermore, recently also in planar Sb_2_S_3_‐based devices short circuit current densities (*J*_SC_) and PCEs of up to 9.7 mA cm^−2^ and 2.5%, respectively, have been reported, which are exceptionally high compared with most other planar systems.^21^ Kim et. al.^19^ demonstrated even more reproducible and highly efficient planar Sb_2_S_3_‐based devices resulting in an *J*_SC_ and PCE of almost 15 mA cm^−2^ and 5.7%, respectively. These high currents are generated by relatively thick (at least for ETA cells) Sb_2_S_3_ layers of high crystal quality. To assure this, the authors employed atomic layer deposition for the synthesis of Sb_2_S_3_, which allows virtually perfect control over the layer growth but is comparably slow (>13 h for a film of 90 nm). Ideally, high efficiency devices have to be realized using low‐cost, upscalable techniques, e.g., solution processing to render them viable for industrialization. These results point toward the importance of crystallinity of the Sb_2_S_3_ layer and are analogous to progress in the perovskite community, where the device performance is strongly dominated by the quality of the perovskite films and their formation process.^30^

This study aims at elucidating the potential of solution‐processed low‐cost Sb_2_S_3_ planar heterojunction devices and the role of the employed HTM. A schematic of the geometry used for solar cell fabrication is shown in Figure 1b.

## 2 Results and Discussion

To exclude effects due to devices with limited functionality, processing procedure and layer thicknesses were first optimized. For this purpose, processing conditions of the CBD, deposition time, and pre‐ and post‐treatments of fabricated Sb_2_S_3_ layers have been varied. This optimization resulted in new record efficiencies of solution‐processed planar Sb_2_S_3_ devices with champion cells exceeding 4% power conversion efficiency utilizing P3HT as HTM and without any surface modification. Even though these values have not been externally certified, great care has been taken to avoid measurement errors as recently discussed in several commentaries.^31^ The best device performance was found for deposition times between 85 and 115 min, leading to compact layer thicknesses of about 50–70 nm with larger crystallites being present on the surface due to the CBD. The role of these crystallites is currently under investigation but was not in the scope of this study. Thinner (85 min CBD time) Sb_2_S_3_ layers resulted in a lower *J*_SC_ of about 9.3 mA cm^−2^ but a higher fill factor (FF) of up to 62% compared with 11.0 mA cm^−2^ *J*_SC_ and 50% FF for thicker (115 min CBD time) Sb_2_S_3_ layers. The open circuit voltage (*V*_OC_) was constant at 0.73 V in both cases. Figure 1c) shows a corresponding current density–voltage (*J*–*V*) curve of one of the most efficient devices. Additionally, the inset in Figure 1c shows the corresponding EQE values of our planar device, normalized to the measured *J*_SC_. While the EQE reaches values of over 80% at short wavelengths of the visible spectrum, it decreased within the absorption spectrum of the HTM indicating a significant parasitic absorption of the HTM. Besides limited Sb_2_S_3_ film thickness, this is identified as the main limitation of the photocurrent in these devices. It is also noteworthy that there is a difference in series resistance between the dark and illuminated curves, which is mainly attributed to a photoinduced filling of deep surface trap states in the TiO_2_ and an additional influence by photoconductivity.^32^, ^33^ Further information on *J*–*V* characteristics and EQE data can be found in Table S1, and Figures S2 and S3 (Supporting Information).

To sort out the contribution of the HTM to these high currents observed under simulated solar light, a more detailed EQE analysis was performed. This includes both nontransparent and semitransparent devices, which were fabricated by evaporating 125 nm and 15 nm Ag, respectively, as back electrodes and measured under three different illumination conditions: (a) nontransparent devices illuminated through the glass side, (b) semitransparent devices illuminated through the glass side (front illuminated), and (c) semitransparent devices illuminated through the back electrode (back illuminated). Then, to identify the HTM contribution, the P3HT layer thickness was varied.

**Figure 2:**
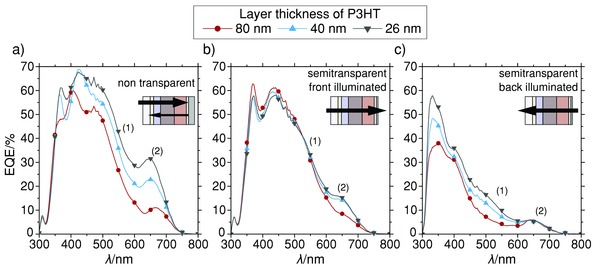
EQE of Sb_2_S_3_‐based planar heterojunction devices for different thicknesses of P3HT and varying illumination condition, revealing 1) the parasitic absorption of the HTM, and 2) a significant optical spacer effect of the HTM. a) Nontransparent devices and b,c) semitransparent devices, illuminated through the glass (front), and through the back‐contact (back), respectively.

**Figure** **2** summarizes experimental data for all illumination conditions and varying layer thicknesses of P3HT, revealing two of the previously mentioned influences of the HTM:

Parasitic absorption: P3HT parasitically absorbs light and significantly lowers the EQE with increasing thickness.

Optical spacer: P3HT acts as an optical spacer^34^ and leads to an additional thickness dependency of the EQE.

The former is apparent from the main absorption region of the polymer (1) around 550 nm (see Figure 2), where the EQE decreases with increasing polymer thickness when the light is passing the polymer layer. This is the case in a) for the nontransparent devices (where the light is back‐reflected from the Ag electrode) and c) back illuminated semitransparent devices. Thereby, the thickness‐independent EQE in b), where no significant amount of light is reflected at the back contact, provides clear evidence that this EQE reduction in region (1) is not caused by charge transport limitations in case of thicker P3HT layers. Additionally, *J*–*V* measurements of these devices confirm an increasing device performance with decreasing HTM layer thickness (see Figure S2, Supporting Information), which further the parasitic absorption by the HTM.

The optical spacer effect, on the other hand, is illustrated at wavelengths above 650 nm (2) (see Figure 2) where P3HT does no longer absorb. There, an increased layer thickness of P3HT causes a shift of the standing electromagnetic wave inside the complete device and results in a changing absorption within the Sb_2_S_3_ layer. This can be observed in a) and b). In contrast, no optical spacer effect is noticeable at (2) for illumination through the back contact because under this illumination condition the standing wave in Sb_2_S_3_ is reduced in intensity with increasing thickness of P3HT but not shifted.

**Figure 3:**
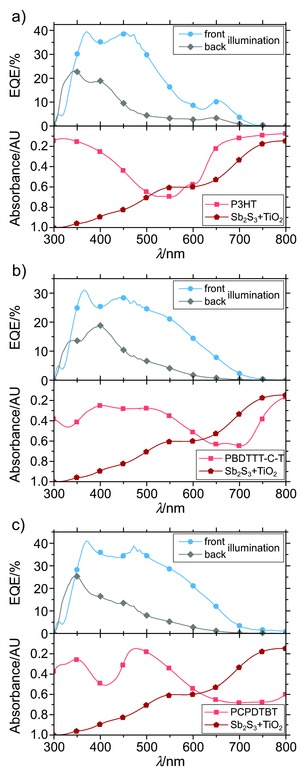
EQE of semitransparent Sb_2_S_3_‐based planar heterojunction devices for different HTMs and varying illumination conditions. Additionally, absorption spectra of corresponding HTMs are shown below the EQE. a) P3HT, b) PBDTTT‐C‐T, and c) PCPDTBT.

Furthermore, low EQE values at point (1) that are missing any spectral connection to the absorption spectrum of P3HT suggest no charge contribution of the HTM. However, since the absorption of P3HT completely overlaps with the absorption of Sb_2_S_3_, this cannot be confirmed with certainty. Thus, measurements on semitransparent devices have been performed also for HTMs absorbing more complementary to Sb_2_S_3_, namely PBDTTT‐C‐T, PCPDTBT. **Figure** **3** shows the experimental EQE results of these additional HTMs in comparison to their corresponding absorption spectrum, suggesting the same parasitic light filtering behavior as for P3HT. Although the effect is not as strong, the parasitic absorption is evident for distinctive absorption peaks of the HTMs in the range between 340 and 400 nm, especially in back illuminated measurements.

Furthermore, the absence of any EQE at wavelengths above 750 nm for PCPDTBT and PBDTTT‐C‐T, where particularly the former shows strong absorption, reaffirms that there is no charge contribution from these HTMs to Sb_2_S_3_. As for P3HT, this is believed to result from an ineffective electron transfer from the polymer^35^ into the Sb_2_S_3_ due to a passivating Sb_2_O_3_ surface layer. This passivation greatly reduces recombination at the interface,^13^ but also can alter the energy level alignment between Sb_2_S_3_ and HTM that leads to less charge transfer states at the interface.^36^

To substantiate our findings, light absorption and charge generation simulations have been performed using the transfer matrix optical modeling simulation by Burkhard et al.^37^ We simulated the light absorption and charge generation of our optimized device with various thicknesses of Sb_2_S_3_ and P3HT, and compared these with the above‐shown experimental data of Figure 1c). To account for the small exciton diffusion length of about 10 nm in P3HT, the P3HT layer was separated into a charge contributing layer (P3HT‐ex) and a bulk layer (P3HT‐bulk).^33^ This leads to the simulated device configuration ITO/TiO_2_/Sb_2_S_3_/P3HT‐ex/P3HT‐bulk/Ag. Starting values of all layer thicknesses have been estimated from scanning electron microscopy (SEM) cross‐sections and numerically optimized within a small range to assure better matching of experimental and simulated data as described in the Supporting Information (see Figures S4 and S5). Finally, to clarify the charge carrier contribution by the HTM the internal quantum efficiency (IQE) of P3HT‐ex has been varied.

**Figure 4:**
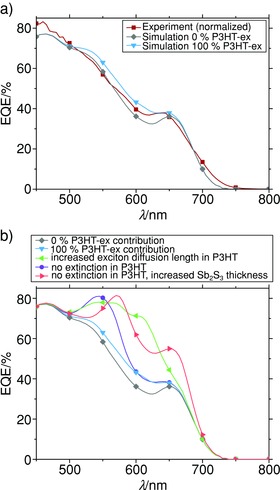
a) Comparison of experimental EQE data of flat Sb_2_S_3_‐based solar cells with best fitting simulations for varying quantum efficiencies of P3HT‐ex. Experimental data were normalized to measure short circuit current density of the corresponding *J*–*V* measurement. b) Hypothetical ways of improvement of Sb_2_S_3_‐based solar cells compared with simulations of a).

**Figure** **4**a shows resulting simulations for 0% and 100% IQE of P3HT‐ex compared with the experimental data of Figure 1c. Thereby, the best fit was found for layer thicknesses of 169 nm ITO, 66 nm TiO_2_, 46 nm P3HT‐bulk, and an average thickness of 155 nm of Sb_2_S_3_, all of which correspond well to experimentally observed layer thicknesses. The average layer thickness of Sb_2_S_3_ is a result of crystalline Sb_2_S_3_ particles covering a large amount of the layer surface (see Figure S6, Supporting Information). The leftover discrepancy between simulated and measured data is attributed to the simplified simulation model of perfectly planar layers, and the used optical data. In particular, spectral ellipsometry measurements of Sb_2_S_3_, and P3HT have been performed on silicon substrates. This can lead to an altered Sb_2_S_3_ growth direction in CBD and thus to slightly different optical properties.^38^ Also, an altered surface alignment of P3HT can lead to a different composition of amorphous and crystalline phase of P3HT and thus result in a shifted absorption spectrum.^39^

Except for slight deviations, simulations are in good accordance with experimental data and reconstruct the parasitic absorption and the optical spacer effect of P3HT‐bulk as dominant influences. However, due to the layered geometry Sb_2_S_3_ absorbs first and significantly lowers the light intensity within the P3HT‐ex layer. This limits a potential charge contribution by P3HT‐ex to 0.5 mA cm^−2^, provided that the whole 10 nm layer contributes with 100% IQE. These small current densities represent only about 5% of measured *J*_SC_ values and are in the same order of magnitude as discrepancies of the simulation, which prevents a certain distinction of charge contribution by P3HT‐ex. To find promising ways of further optimization, additional simulations have been performed for various P3HT and Sb_2_S_3_ conditions. Figure 4b summarizes hypothetical ways of improvement for the layer thickness constellation in Figure 4a. In particular, an increase in exciton diffusion length in P3HT, the elimination of its extinction, and an increase in Sb_2_S_3_ layer thickness have been tested. While an increase in exciton diffusion length in P3HT is experimentally hard to realize, alternative HTMs could be employed in the future that exhibit this property. The benefits in charge generation could result in a significantly enhanced charge carrier contribution of 30% by the complete 56 nm thick HTM layer (P3HT‐ex + P3HT‐bulk) instead of 5% (P3HT‐ex) in the current situation. Elimination of the extinction coefficient of P3HT could result in an improvement of 14% in *J*_SC_. In EQE the loss of any P3HT‐ex contribution is compensated and surpassed by an additionally gained absorption in Sb_2_S_3_. This can be further optimized to 30% by increasing the layer thickness of Sb_2_S_3_ to 250 nm. Even though good Sb_2_S_3_ crystallinity is challenging to realize using only low‐temperature solution processing, our results point toward the possibility of fabricating such devices when further optimizing the CBD.

## 3 Conclusions

In summary, we have fabricated planar, solution processed Sb_2_S_3_‐based hybrid solar cells with efficiencies exceeding 4%. We investigated the influence of different commonly used hole transport materials in combination with Sb_2_S_3_. As a result, the HTM does not seem to contribute significantly to charge generation, and the characteristic EQE shape is caused by the absorption spectrum of Sb_2_S_3_ in combination with the parasitic absorption of the HTM. However, even with a probably high contribution of the polymer to charge generation within the exciton diffusion length of around 10 nm, the parasitic absorption of the HTM would dominate the influence on device performance. To further improve device efficiency, commonly used HTMs have to be replaced by materials with higher exciton diffusion lengths as for instance highly crystalline small‐molecular layers^40^ to maximize HTM photocurrent contribution. Alternatively, and with a slightly lower positive effect, high band gap materials can be employed, which should exhibit good hole transport properties and have to be optimized in terms of layer thicknesses for a beneficial optical spacer effect. CuSCN has shown to be a promising candidate as HTM since it shows required optical and electronic properties, and additionally forms a good band alignment to Sb_2_S_3_. However, recombination at the interface is still a limiting factor leading to small open circuit voltages in fabricated devices. Alternatively, the small molecule HTM spiro‐OMeTAD shows a suitable band alignment to Sb_2_S_3_, and a band gap of about 3 eV.^41^ Nevertheless, both of these wide band gap hole transporters have proven less efficient than polymers such as P3HT or PCPDTBT,^2^, ^13^ demanding for the development of an alternative new hole transporter for future high‐efficiency Sb_2_S_3_ devices.

## 4 Experimental Section

*Materials*: Precursors (Na_2_S_2_O_3_ and SbCl_3_) for chemical bath deposition of Sb_2_S_3_ were purchased from Sigma‐Aldrich. P3HT was purchased from Rieke Metals or Merck, and PCPDTBT and PBDTTT‐C‐T were purchased from 1‐Materials and Solarmer Materials Inc., respectively. All other chemicals were purchased from Sigma‐Aldrich in high purity grade (>99%). All chemicals were used without further purification.

*Optimized Solar Cell Preparation*: Tin‐doped indium oxide (ITO) glass substrates (Lumtec, <10 Ω/□) were subsequently cleaned for 5 min each in ultrasonic baths of deionized water with commercial dishwashing detergent, acetone, and isopropyl alcohol (IPA). A compact TiO_2_ layer was deposited from a 1:10 solution of titanium diisopropoxide bis(acetylacetonate) in ethanol via spray pyrolysis at 450 °C. Therefore, substrates were annealed from room temperature to 450 °C within 15 min, held at this temperature for 15 min, and slowly cooled to 120 °C. The Sb_2_S_3_ layer is prepared by chemical bath deposition (CBD) according to a previously reported procedure with minor adjustments.^4^ In particular, solutions of Na_2_S_2_O_3_ (4 g) in deionized water (25 mL) and SbCl_3_ (650 mg) in acetone (2.5 mL) were prepared and cooled in an ice bath for 90 min. Precursors were mixed into 100 mL of likewise cooled deionized water and samples are immediately put into the mixed solution. After chemical bath deposition samples were rinsed with deionized water and promptly dried in a nitrogen stream. Subsequently, the coated backside of the samples was cleaned with hydrochloric acid, and the samples were annealed at 300 °C for 35 min in a nitrogen atmosphere. The P3HT solution (Rieke Metals, 15 mg mL^−1^ in chlorobenzene (CB)) was then spin‐coated at 1500 rpm for 1 min. As a top contact, 150 nm Ag was deposited via thermal evaporation at deposition rates of 0.1–2 Å s^−1^.

*Semitransparent Solar Cell Preparation*: Concentrations of P3HT solutions for devices in Figure 2 with layer thicknesses of 80, 40, and 26 nm, respectively, were 20, 10, and 6.7 mg mL^−1^ in CB. Concentrations of the different hole transport materials for devices in Figure 3 were 20 mg mL^−1^ P3HT, 15 mg mL^−1^ PCPDTBT, and 20 mg mL^−1^ PBDTTT‐C‐T. All HTMs were spin‐coated at 2000 rpm for 1 min. Additionally, 8 nm WO_3_ was evaporated in between HTM and Ag back contact. For semitransparent devices, the evaporated Ag back contact was only 15 nm.

*Reproducibility Issues of the CBD*: Fine‐tuning of the deposition and post‐deposition processes was extremely important for achieving high efficiencies above 3% PCE. In particular, during deposition samples were placed tilted (face down) into the bath to avoid large particles attach to the deposition layer. After deposition, rinsing and drying of substrates have to be done quickly, since layers are easily detaching from the TiO_2_ surface during prolonged contact with deionized water. After cleaning, substrates were transferred to a nitrogen‐filled glove box within 30 min and directly annealed to 300 °C, with a ramp time of about 7 min. Subsequently, the samples were slowly cooled to room temperature. Additionally, reproducibility and layer quality also depend on ambient conditions as temperature and humidity.

*Absorption Measurements*: Absorption of fabricated layers of Figure 3 has been measured with an Agilent 8453 UV–vis spectrophotometer, whereas collected data have been obtained in optical density (OD) without correction for scattering and reflection. For a better comparison, data have been recalculated to absorbance (AU) by $\mathrm{Abs}=1-10^{-\mathrm{OD}}$. Original data are shown in Figure S7 (Supporting Information). Arbitrary units were chosen due to missing correction for scattering and reflection.

*J–V Measurements*: Solar cells were illuminated with an AM 1.5G solar simulator and the light intensity adjusted to 100 mW cm^−2^ using a Fraunhofer ISE certified Si reference diode with a KG5 filter. *J*–*V* curves were acquired with a Keithley 2400 sourcemeter controlled by a LabView program. Solar cells were placed in a light tight sample holder (to avoid additional excitation of the materials due to scattered light) and illuminated through a shadow mask defining three times an active area of 0.125 cm^2^ for the three pixels on each substrate.

*EQE Measurements*: EQE was measured using the same solar cell holder and shadow mask as for *J*–*V* measurements. As an illumination source, a 150 W Xe lamp was used in combination with a monochromator. Monochromatic light was focused on the solar cell with a spot size larger than the active area of the device. Since our setup performs EQE measurements without a white light background, acquired spectra of best performing devices in Figures 1c and 4 were normalized to the short circuit current density of each corresponding cell under 100 mW cm^−2^ solar illumination to avoid overestimation of the EQE. Corresponding unnormalized data can be found in Figure S2 (Supporting Information). Figures 2 and 3 already represent unnormalized EQE data.

*Simulation*: Simulations of light intensity distribution, absorption, and charge generation within our device configuration were done with an adjusted version of the transfer matrix optical modeling script by Burkhard et al.^37^ (downloaded from http://web.stanford.edu/group/mcgehee/transfermatrix/). Field patterns have been calculated for wavelengths between 450 and 800 nm, and the lattice point distance for the calculation of the electric field was taken as 1 nm. Internal quantum efficiencies of TiO_2_ and Sb_2_S_3_ have been set to 10 and 90%, respectively. Fit of layer thicknesses was done for all possible combinations at once, and subsequently selected by the method of least squares, whereas the normalized EQE spectra were taken as reference. Dispersion relations of refractive indices and extinction coefficients of ITO and silver have been taken from the database attached to the simulation program package. Values of anatase TiO_2_ by Schubert^42^ were downloaded from http://homepages.rpi.edu/~schubert/, whereas the values of P3HT and Sb_2_S_3_ have been determined and fitted by spectral ellipsometry and interpolated with a cubic spline function (see Figure S1, Supporting Information).

## Supporting information

As a service to our authors and readers, this journal provides supporting information supplied by the authors. Such materials are peer reviewed and may be re‐organized for online delivery, but are not copy‐edited or typeset. Technical support issues arising from supporting information (other than missing files) should be addressed to the authors.

Supplementary
