# Supplementary material for: Toward High‐Efficiency Solution‐Processed Planar Heterojunction Sb2S3 Solar Cells
Source: Adv Sci (Weinh). 2015 Apr 2;2(5):1500059. doi: 10.1002/advs.201500059 (PMC5115364; doi:10.1002/advs.201500059)
Supplement: Supplementary file 1 — Supplementary [file ADVS-2-0n-s001.pdf]

Copyright WILEY-VCH Verlag GmbH & Co. KGaA, 69469 Weinheim, Germany, 2015.

ADVANCED SCIENCE
Open Access

Supporting Information

for *Adv. Sci.*, DOI: 10.1002/advs.201500059

Toward High-Efficiency Solution-Processed Planar Heterojunction Sb_2S_3 Solar Cells

Eugen Zimmermann, Thomas Pfadler, Julian Kalb, James A. Dorman, Daniel Sommer, Giso Hahn, Jonas Weickert, and Lukas Schmidt-Mende*

((Supporting Information can be included here using this template))

Copyright WILEY-VCH Verlag GmbH & Co. KGaA, 69469 Weinheim, Germany, 2013.

Supporting Information

Towards High-Efficiency Solution-Processed Planar Heterojunction Sb_2S_3 Solar Cells

Eugen Zimmermann, Thomas Pfadler, Julian Kalb, James A. Dorman, Daniel Sommer, Giso Hahn, Jonas Weickert, Lukas Schmidt-Mende*

Dispersion relations of TiO_2, Sb_2S_3, and P3HT

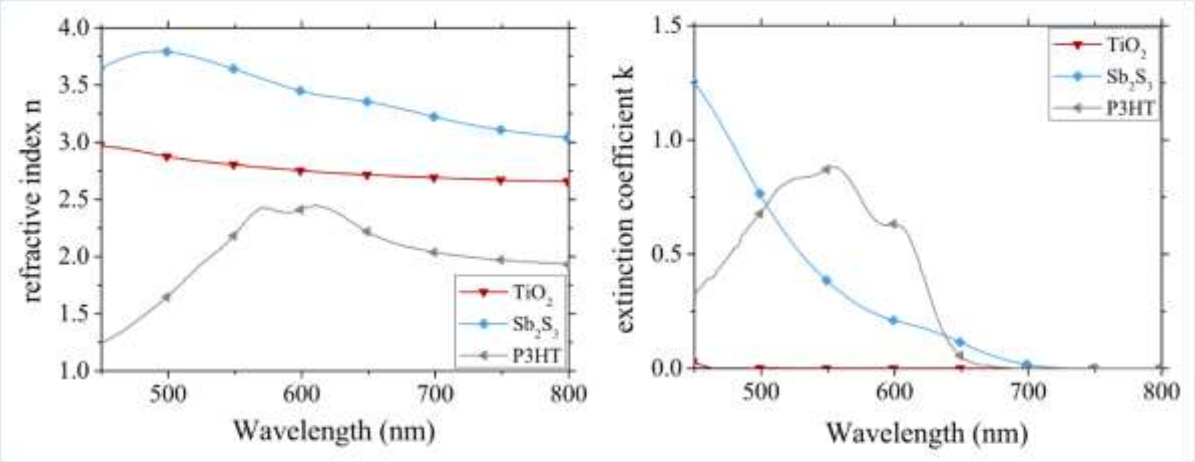

Figure S1. Refractive indices and extinction coefficients of investigated materials TiO_2, Sb_2S_3, and P3HT. Ellipsometry measurements have been performed for values of Sb_2S_3 and P3HT, whereas values of anatase TiO_2 were taken from literature.[42]

Solar Cell Performance

Table S1 summarizes JV measurement parameters and Figure S2 shows EQE measurements of fabricated best performing solar cell devices with two different chemical bath deposition times of Sb_2S_3 and P3HT as HTM. Figure S3 shows the foregoing optimization process of another solar cell batch with varying P3HT layer thickness. All solar cells of each particular Table or Figure are fabricated within one batch in one experiment to assure direct comparability of the results.

Table S1. J-V characteristics of record efficiency flat Sb_2S_3 based solar cells with two different chemical bath deposition times and P3HT as HTM.

Cell #	***Sb_2S_3 CBD time (min)***	**PCE (%)**	**V_{OC} (mV)**	**J_{SC} (mA/cm^2)**	**FF (%)**
**1**	**85**	3.93	727	8.96	60
**2**	**85**	4.25	732	9.34	62
**3**	**115**	4.06	726	11.01	50
**4**	**115**	3.81	714	10.95	48

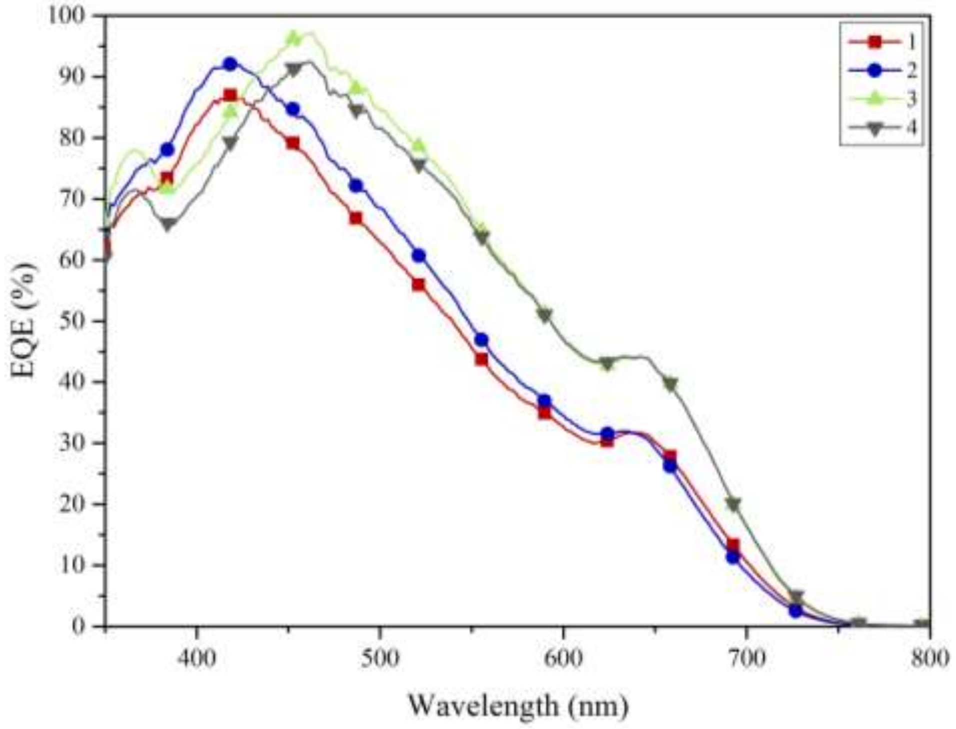

Figure S2. Corresponding unnormalized EQE of optimized Sb_2S_3 based solar cells with varying layer thicknesses of Sb_2S_3.

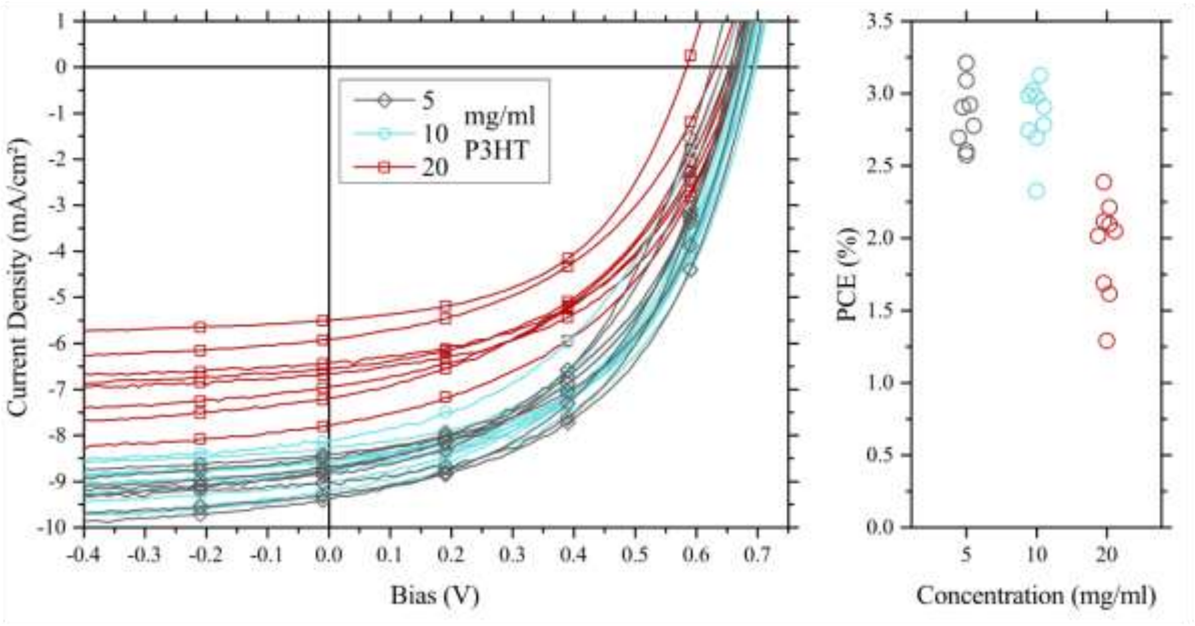

Figure S3. JV characteristics of foregoing optimization of flat Sb_2S_3 based solar cells with different P3HT layer thicknesses. Layer thicknesses were adjusted by changing the concentration of P3HT and resulting film thicknesses were measured by atomic force microscopy.

Scanning Electron Microscopy Characterization

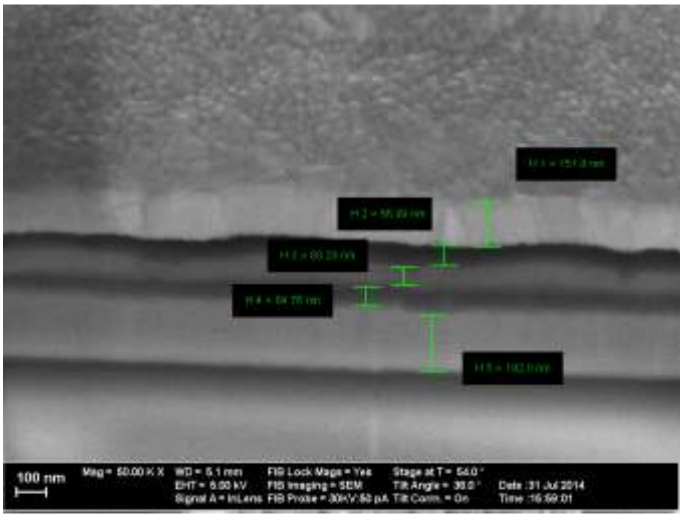

Figure S4. Cross section of Device 3 of Figure S2, and Table S1 in the SEM, cut by focused ion beam.

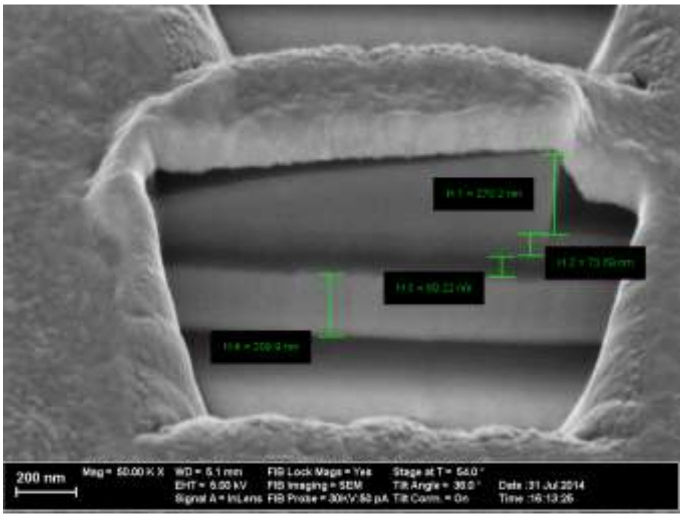

Figure S5. Cross section of Device 3 of Figure S2, and Table S1 in the SEM, cut through a large Sb_2S_3 crystal by focused ion beam.

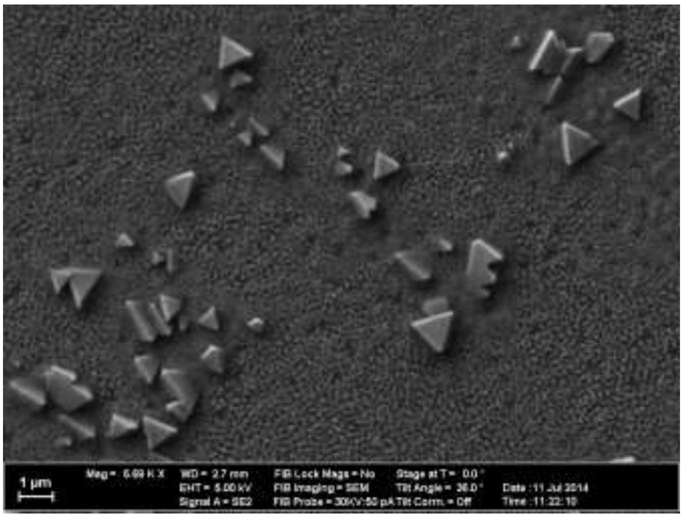

Figure S6. Scanning electron microscopy image of the surface of Device 3, shown in Figure 1 c) and S2, and Table S1.

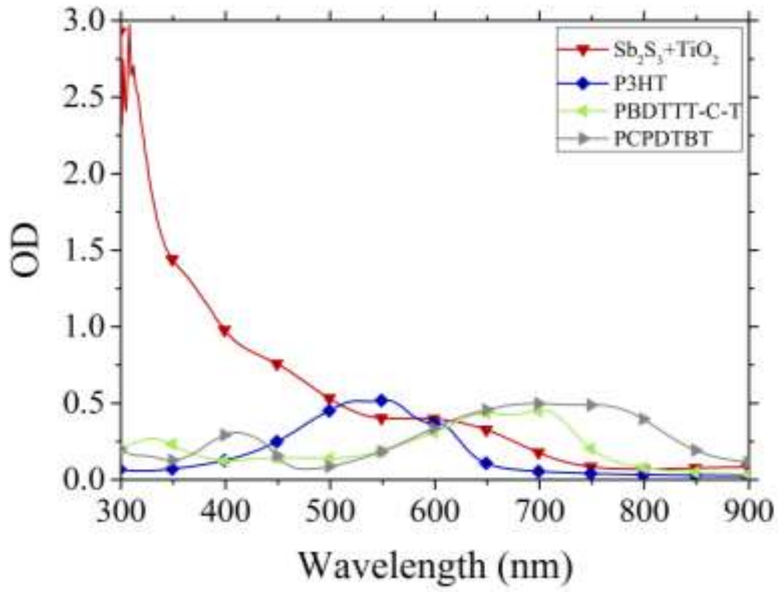

Figure S7. Absorption spectra of Sb_2S_3 and used HTMs.
